# First Mnks degrading agents block phosphorylation of eIF4E, induce apoptosis, inhibit cell growth, migration and invasion in triple negative and Her2-overexpressing breast cancer cell lines

**DOI:** 10.18632/oncotarget.1528

**Published:** 2014-01-25

**Authors:** Senthilmurugan Ramalingam, Lalji Gediya, Andrew K. Kwegyir-Afful, Vidya P. Ramamurthy, Puranik Purushottamachar, Hannah Mbatia, Vincent C. O. Njar

**Affiliations:** ^1^ Department of Pharmacology, University of Maryland School of Medicine, Baltimore, MD, USA; ^2^ Center for Biomolecular Therapeutics, University of Maryland School of Medicine, Baltimore, MD, USA; ^3^ Marlene Stewart Greenebaum Cancer Center, University of Maryland School of Medicine, Baltimore, MD, USA

**Keywords:** Breast cancer, RAMBA retinamides, eukaryotic initiation factor 4E, Mnk1/2

## Abstract

Some retinoic acid metabolism blocking agents (RAMBAs) are known to exhibit a wide range of anticancer activities by mechanisms that are still not completely resolved. This study investigated the anticancer efficacy and mechanism(s) of novel RAMBA retinamides (RRs) in triple negative and Her-2 overexpressing breast cancer cells. Specifically, we examined the possibility that RRs affect the translational machinery in these breast cancer (BC) cells. Recent findings suggest that overexpression of eukaryotic translation initiation factor 4E (eIF4E) in breast cancers critically augments CAP-dependent mRNA translation and synthesis of proteins involved in cell growth, cell proliferation, invasion and apoptosis evasion. The oncogenic potential of eIF4E is strictly dependent on serine209 phosphorylation by upstream MAPK-interacting kinases (Mnks). Targeting Mnk/eIF4E pathway for blocking Mnk function and eIF4E phosphorylation is therefore a novel approach for treating BCs, particularly for Her2-positive and triple negative breast cancers that have no indications for endocrine therapy or effective treatment regimes. We report for the first time that the degradation of Mnk1 by RRs in BC cells blocks eIF4E phosphorylation and subsequently inhibits cell growth, colonization, invasion, and migration and induce apoptosis. Most importantly, the anticancer efficacy of RRs was mediated via degrading Mnk rather than inhibiting its kinase activity like Mnk inhibitors (cercosporamide and CGP57380). Furthermore, RRs potencies on peIF4E down-regulation and growth inhibition were superior to those of two clinically relevant retinoids and the Mnk inhibitors. Together our findings provide the first preclinical proof-of-concept of novel Mnk degrading agents for Mnk/eIF4E based therapeutic treatment of breast cancers.

## INTRODUCTION

Despite significant advances toward targeted therapy and screening techniques, breast cancer remains the most frequently diagnosed female malignancy and leading cause of cancer-related deaths worldwide [1]. Approximately 60–70% of breast cancers are hormone receptor positive expressing estrogen (ER) and/or progesterone receptors (PR). Selective amplification and/or overexpression of human epidermal growth factor receptor 2 (Her2) is found in approximately 25–30% of all primary breast cancers. Her2 plays a critical role in regulating cell proliferation, adhesion, motility, and survival, and Her2 overexpression results in aggressive tumor behavior, clinical resistance and poor prognosis [2]. In addition, 20–30% of breast cancers are triple-negative breast cancers (TNBC), lacking ER, PR and Her2 with more aggressive clinical course than other forms of breast cancer, increased local/systemic relapse and poor survival [2–4]. Identifying novel molecular drug targets for TNBCs that have no indications for endocrine therapy, and more efficacious therapy for Her2-positive breast cancer has therefore become extremely imperative in developing effective treatment strategies for breast cancer patients. Several breast cancer studies have identified numerous genes such as p53, PIK3CA, AKT1, PTEN, and EGFR to be commonly mutated in breast cancers. Activating mutations in these genes promote aberrant activation of certain signaling cascades and in turn tumor growth via up-regulation of key oncogenic proteins [2].

Recent progress in molecular pathogenesis of cancer has identified eukaryotic translation initiation factor 4E (eIF4E), an oncogenic rate-limiting factor of cap-dependent translation to play a critical role in the translation of certain weak mRNAs such as cyclin D1, Bcl-2, MMPs and VEGF that are vital for oncogenic transformation [5–9]. eIF4E, regulated by mitogen-activated protein kinase (MAPK)/Mnk and phosphoinositide 3-kinase-protein kinase B-mammalian target of rapamycin (PI3K/Akt/mTOR) signaling pathways facilitates efficient initiation of mRNA translation by binding to the 5′-cap structure of eukaryotic mRNAs [10, 11]. Studies with eIF4E expressing transgenic mice have revealed a marked increase in the incidence of various cancers including lymphomas, lung adenocarcinomas, angiosarcomas, and hepatomas [12]. An association between increased eIF4E expression and cellular transformation is also seen in several tumors including that of the breast, bladder, colon, head and neck, lymphoma, lung, and thyroid [13]. Increased levels of eIF4E and activated translation initiation are also considered crucial for breast cancer progression and angiogenesis [14]. eIF4E overexpression is further regarded as a poor prognostic marker for breast cancer [13, 15–18]. Several approaches for impeding eIF4E dependent translation initiation have been conceived in recent years including the use of eIF4E targeted antisense oligonucleotide (ASO), antiviral guanosine analogues, small molecule inhibitors and natural phytochemicals such as curcumin and silibinin [19–22]. These data collectively suggest that targeting MAPK/Mnk signaling and blocking eIF4E protein translational machinery to be a promising strategy for treating TNBCs and other breast cancers [17, 23].

Recently, a family of retinoic acid metabolism blocking agents (RAMBAs) described by us that chiefly inhibit cytochrome P450 enzymes responsible for the metabolism of all-*trans*-retinoic acid (ATRA) was show to exert potent anticancer and growth inhibitory effects in human breast/prostate cancer cells and breast/prostate cancer xenograft models [24–30]. Several novel potent RAMBAs that are structural analogues of ATRA and 4-hydroxyphenyl retinamide (4-HPR or fenretinide) have been designed and synthesized in our laboratory [27, 28]. Previous studies have shown that these RAMBAs inhibit cell proliferation and induce intrinsic apoptosis by decreasing cyclin D1 and up-regulating pro-apoptotic proteins [24, 25, 28, 30, 31]. Of particular interest is that among the proteins regulated by translation initiator, eIF4E, a number of them, such as cyclin D1, Bax, Bad and Bcl-2 are also modulated by our RAMBAs [24, 30]. Recent studies have suggested that activation of eIF4E is particularly important for the translation of a subset of cancer promoting mRNAs including cyclin D1 and anti-apoptotic proteins [8, 19, 32]. These observations led us to postulate that RAMB A retinamides (RRs) could mediate their inhibitory anticancer effects in part by modulating the eIF4E translational machinery.

This study was undertaken to evaluate the effects of RRs on growth inhibition, MAPK/Mnk mediated eIF4E protein translational machinery and downstream biological effects in triple negative and Her2 overexpressing breast cancer cells. Our data demonstrate that RRs are capable of degrading Mnk proteins, blocking eIF4E phosphorylation, modulating cell cycle proteins thereby inhibiting cell proliferation and survival, reducing colony formation, inducing intrinsic apoptosis and impeding tumor invasion and migration in breast cancer cells. The present study therefore authenticates a novel function for RRs in degrading Mnk and blocking eIF4E dependent translational initiation, and further endorses the notion that RRs are novel potential therapeutic agents for treating triple negative and Her2 overexpressing breast cancers.

## RESULTS

### RRs inhibit cell proliferation and colony formation of aggressive human breast cancer cells

**Figure 1:**
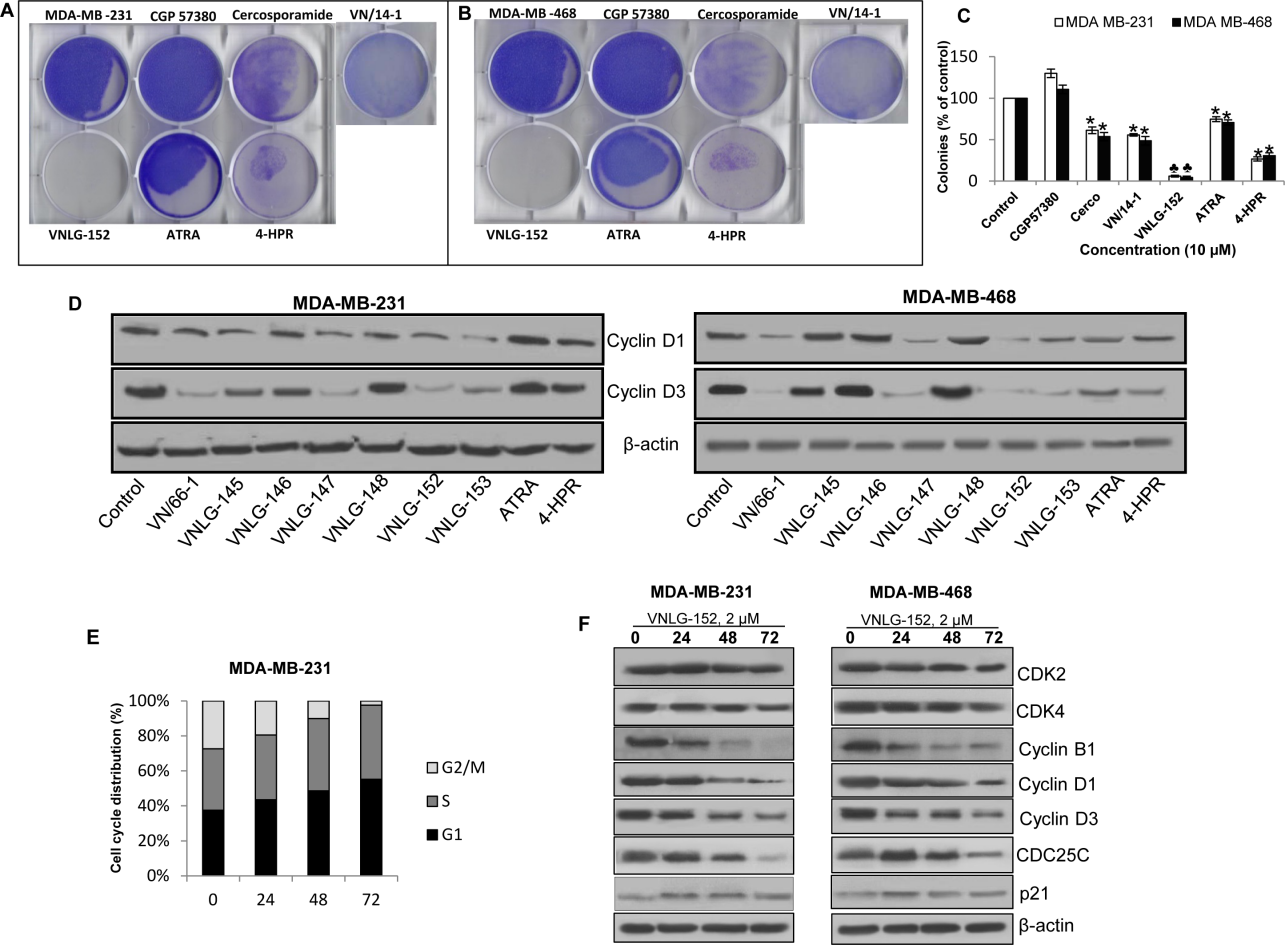
Effect of VNLG-152 on anchorage-dependent growth and cell cycle distribution MDA-MB-231 (A) and MDA-MB-468 (B) cells were treated with VNLG-152, ATRA, 4-HPR, VN/14-1 and Mnk inhibitor (10 μmol/L) for 14 days. Colonies were fixed with methanol and stained with crystal violet. (C) Data represents the mean ± S.E from three independent experiments. *, *P*< 0.01; ♣, *P*<0.001 compared with vehicle treated control. (D) MDA-MB-231 and MDA-MB-468 cells were treated with indicated compound 15 μmol/L for 24 h. Total cell lysates were separated by SDS-PAGE and probed with cyclin D1 and D3 antibodies. (E) MDA-MB-231 cells were treated with VNLG-152 (2 μmol/L) for indicated time period, stained with PI and analysed with a FACScalibur flow cytometer. (F) Western blotting for cell cycle related proteins. Vehicle treated cells were included as a control and all blots were reprobed for β-actin for equal protein loading and transfer.

We first investigated the effect of novel RRs [VN/14-1 (parent RRs), VN/66-1, VNLG-145, −146, −147, −148, −152, −153] and Mnk inhibitors (CGP57380 and cercosporamide) on the growth and proliferation of triple negative (MDA-MB-231 and MDA-MB-468) and Her-2 overexpressing (SKBR-3) breast cancer cells by MTT and colony formation assays. MTT results revealed that RRs, in particular VNLG-147,−152 and −153 potently inhibited the growth of MDA-MB-231 and MDA-MB-468 cells with GI_50_ values ≤ 2 μmol/L and SKBR-3 cells with GI_50_ values ≤ 4 μmol/L (Table 1). Identical results were also obtained in ERa positive MCF-7 breast cancer cells for the lead compound (VNLG-147, −152 and −153). The GI_50_ values for these compounds are ≤ 2 μmol/L (Supplementary Fig. 1A). The GI_50_ values (1.28 – 3.54 μmol/L) of most potent RRs were more potent that the GI_50_ values (26.02 – 96.45 μmol/L) of the Mnk inhibitors. Lead RR (VNLG-152) was also found to significantly reduce MDA-MB-231 and MDA-MB-468 colony size and number. Other RRs, ATRA, 4-HPR and Mnk inhibitors not only displayed reduced potency in inhibiting cell viability but also did not have any profound effects in inhibiting colony formation (Table 1, Fig. 1A, B and C). Importantly, immortalized non-cancerous MCF10A breast cell line was less sensitive to lead RR, VNLG-152 than the other cancer cell lines (Supplementary Fig. 1B). Besides no significant reduction in MCF10A colony size and number was observed upon VNLG-152 treatment (Supplementary Fig. 1C and D).

**Table 1:** Antiproliferative potencies of RRs, ATRA, 4-HPR and Mnk inhibitors in breast cancer cell lines

Compounds*	GI50 values for 2.5 × 10^3^ MDA-MB-231 (μmol/L)	GI_50_ values for 2.5 × 10^3^ MDA-MB-468 (μmol/L)	GI_50_ values for 2.5 × 10^3^ SKBR-3 (μmol/L)
**VN/14-1**	42.65	57.54	32.56
**VN/66-1**	1.86	8.31	3.46
**VNLG-145**	2.88	4.89	3.54
**VNLG-146**	10.23	39.81	12.88
**VNLG-147**	1.41	1.99	3.54
**VNLG-148**	5.75	30.19	19.05
**VNLG-152**	1.44	1.28	3.38
**VNLG-153**	2.00	2.23	2.45
**CGP57380**	36.30	96.45	29.58
**Cercosporamide**	43.65	47.86	26.02
**ATRA**	14.12	14.12	22.90
**4-HPR**	3.39	8.91	2.95

**Note:** Cells were treated with listed compound (0.1 nmol/L-100 μmol/L) for 6 d and the GI_50_ values for the antiproliferative effects of the compounds were determined from dose response curves (by a nonlinear regression analysis using Graph Pad Prism). Data represents the results from six independent experiments for each cell line

* **For clarity, the structures of the compounds are presented below:**

**Figure:**
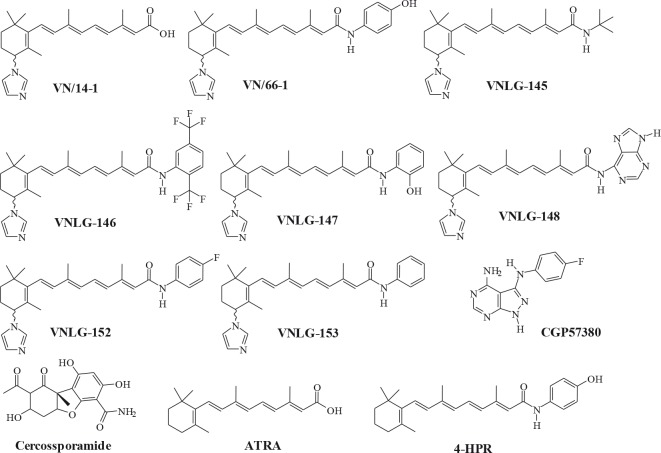

To gain further insight into the mechanism by which RRs induces cell growth inhibition, we studied the effect of RRs on cell cycle regulatory proteins (cyclins D1 and D3) in MDA-MB-231 and MDA-MB-468 cells. We found that 24 h treatment of RRs resulted in a marked decrease in the expression of both cyclin D1 and cyclin D3 (Fig. 1D). To understand how VNLG-152 inhibits cell proliferation by downregulating cyclins, cell cycle analysis was performed. As seen in Fig. 1E, treatment with VNLG-152 (2 μM) up to 72 h induced a time dependent cell cycle arrest at G0/G1 and S phase in MDA-MB-231 cells. The concentration of VNLG-152 required to induce cell cycle arrest is consistent with the GI_50_ value of VNLG-152 required to reduce TNBC cell viability (Table 1). The G0/G1 and S phase arrest induced by VNLG-152 was associated with downregulation of cyclins D1, D3, B1 and CDC25C that are involved in G0/G1 and S phase transition. Furthermore expression of CDK2 and CDK4 were slightly downregulated and p21, a CDK inhibitor was slightly upregulated (Figure 1F). The down-regulatory effects of RRs on cyclins are consistent with our earlier reports on cyclin D1 down-regulation by RAMBAs [24, 30].

### RRs induce apoptosis to inhibit breast cancer cell growth

**Figure 2:**
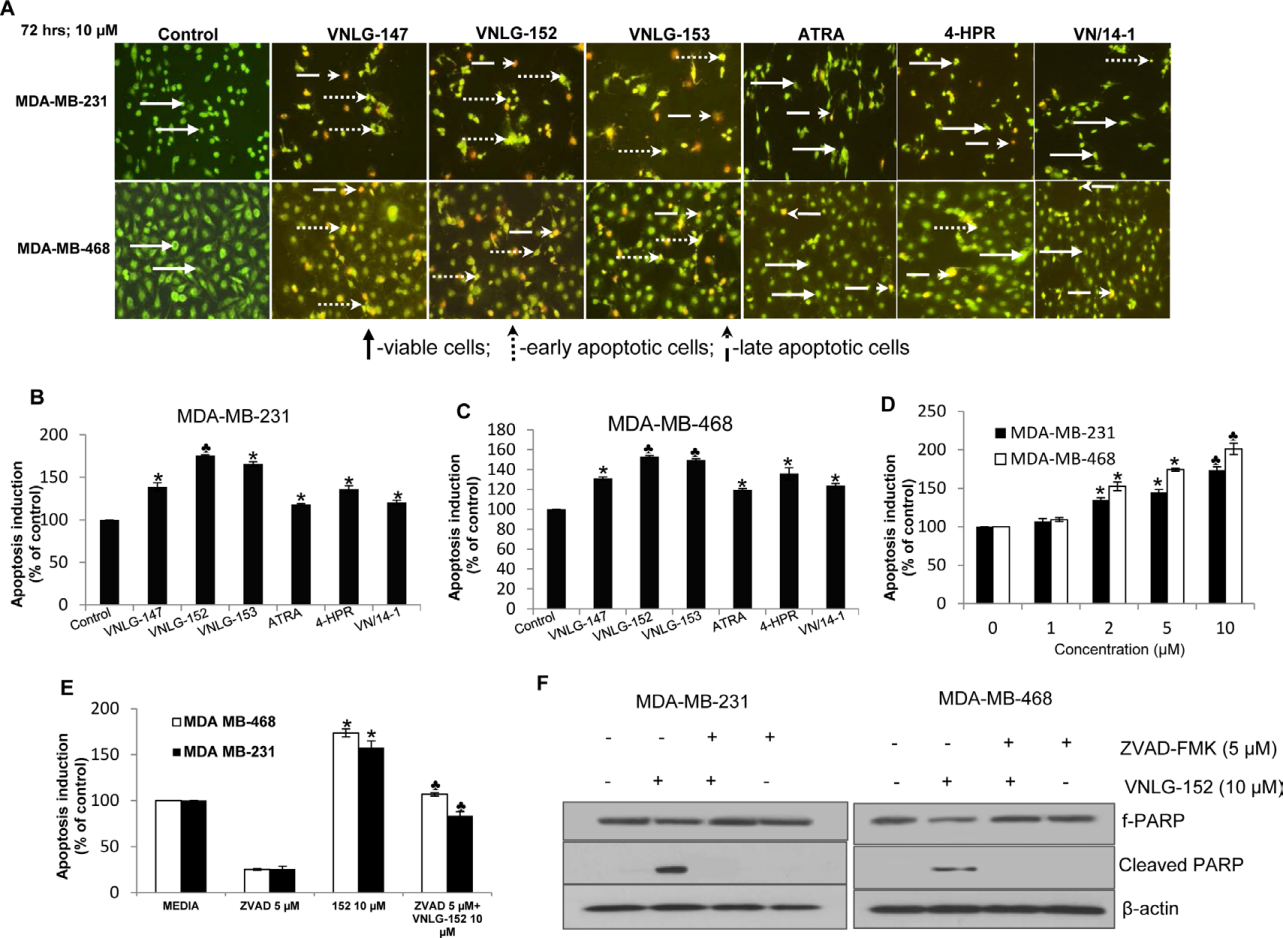
RRs induces apoptosis in TNBC cells (A) The individual cell lines as indicated were seeded in 24 well plate, and treated with 10 μmol/L of VNLG-152 the next day. After 72 h the plates were analyzed for early apoptotic and viable cells using acridine orange/ethidium bromide staining. (B & C) Indicated cells were treated with VNLG-147, -152, -153 ATRA, 4-HPR and VN/14-1 (10 μmol/L) for 72 h and apoptosis induction was examined by oligonucleosomal fragmentation. Data are shown relative to vehicle treated control and the bars are means of three replicate determinations plus standard deviations. *, *P*< 0.05; ♣, *P*<0.01 compared with vehicle treated control. (D) Apoptosis induction in TNBC cells by VNLG-152 (1–10 μmol/L) was assessed by oligonucleosomal fragmentation after a 72 h incubation. (E) Apoptosis induction in triple negative breast cancer cells by VNLG-152 (10 μmol/L) was evaluated with or without the caspase inhibitor ZVAD (5 μmol/L). Data are shown relative to vehicle treated control and the bars are means of three replicate determinations plus standard deviations. *, *P*< 0.01, control versus VNLG-152; ♣, *P*<.01, ZVAD versus ZVAD plus VNLG-152. (F) Expression of PARP and cleaved PARP was investigated after treatment with VNLG-152 (10 μmol/L) and with or without the caspase inhibitor ZVAD (5 μmol/L) by western blot.

To further determine whether the observed decrease in cell numbers was due to induction of cell death, we evaluated the apoptosis inducing potential of lead RRs in TNBC cells by acridine orange/ethidium bromide (AO/EB) dual staining and cell death detection ELISA assay (Roche Diagnostics). AO/EB dual staining established that VNLG-152 (10 μM, 72 h) exhibit strong pro-apoptotic effects compared to that of other RRs and ATRA or 4-HPR (10 μM, 72 h) (Fig. 2A). The apoptosis inducing potential of the lead RRs was also confirmed by cell death detection ELISA assay (Fig. 2B and C). In both the TNBC cells, VNLG-152 significantly induced apoptosis at concentration starting with 2 μM (Fig. 2D). This concentration is consistent with the GI_50_ value of VNLG-152 required to reduce TNBC cell viability (Table 1). To further assess whether VNLG-152 induced apoptotic cell death is dependent upon caspases, we co-treated TNBC cells with VNLG-152 and the caspase inhibitor ZVAD (5 μM, 72 h). Our results demonstrated that combined treatment of ZVAD completely suppressed VNLG-152 induced oligonucleosomal fragmentation, thereby confirming that VNLG-152 induced apoptosis via the caspase-dependent pathway (Fig. 2E). Co-treatment of VNLG-152 and ZVAD also completely blocked VNLG-152 induced PARP cleavage, a distinct feature of apoptosis in TNBC cells (Fig. 2F).

### RRs exerts strong anti-migratory and anti-invasive effects in breast cancer cells

**Figure 3:**
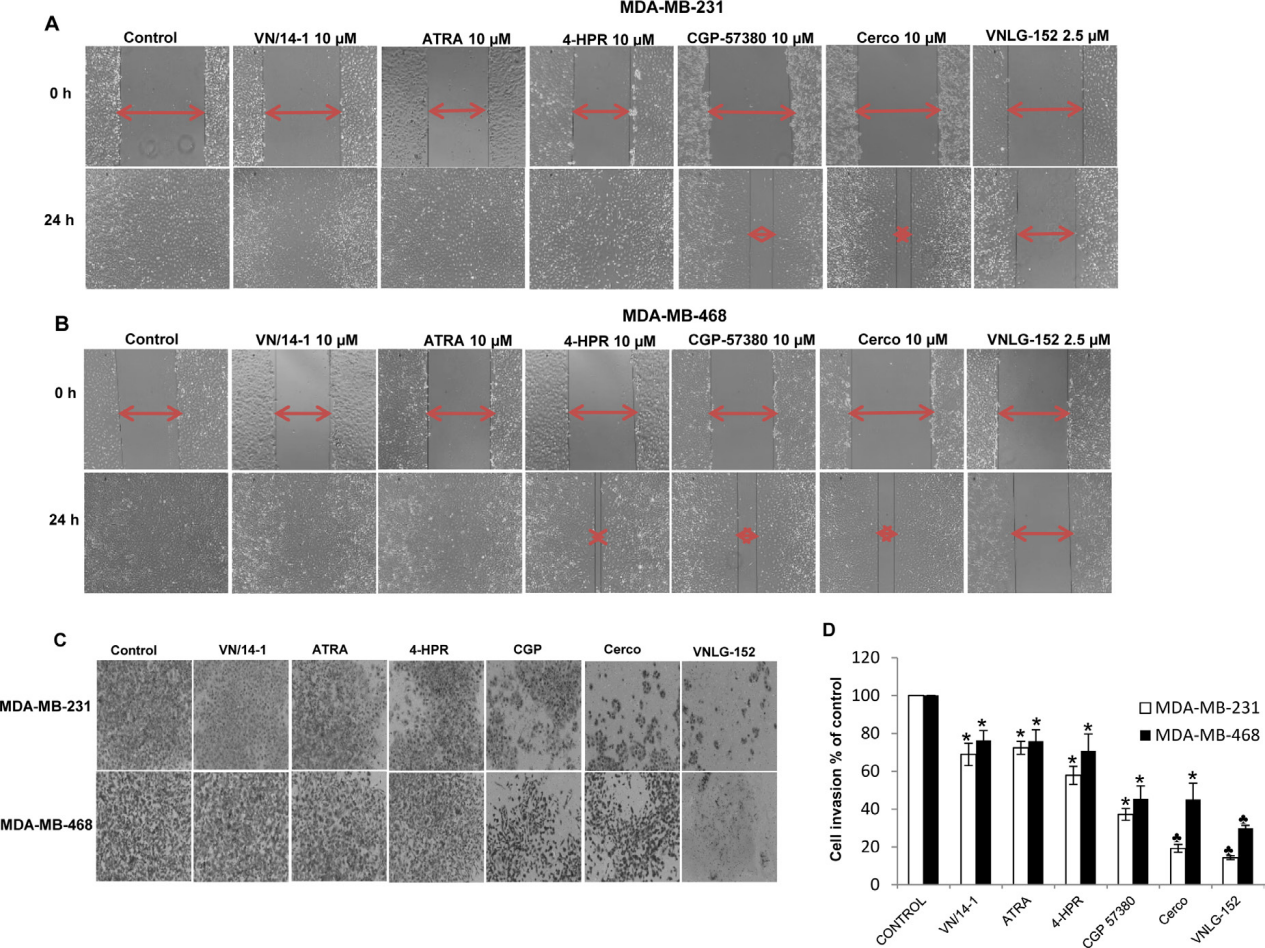
VNLG-152 inhibits migratory and invasive potential of TNBC cells (A & B) Effect of the treatment of VNLG-152 (2.5 μmol/L) and indicated compounds (10 μmol/L) on breast cancer cell migration by wound healing assay for 24 h. (C) Cells were seeded on matrigel coated boyden chamber and treated with VNLG-152 (2 μmol/L) and indicated compounds (10 μmol/L). Dose selection was based on dose-dependent studies. Representative photomicrographs of initial and final wounds and membrane invasion are shown at 100x magnification. (D) Quantification of the number of invaded cells and the data are shown relative to vehicle treated control and the bars are means of three replicate determinations plus standard deviations. *, *P*< 0.01; ♣, *P*<0.001 compared with vehicle treated control.

Invasion is an imperative step in breast cancer cell metastasis [37]. We therefore next evaluated the effects of RRs on the anti-migratory and anti-invasive potential of MDA-MB-231 and MDA-MB-468 cells using wound-healing, PET membrane migration and matrigel invasion assays. We found that 24h after well monolayers were wounded, control cells completely filled the scratched area. Treatment with VNLG-152 (2.5 μmol/L) potentially inhibited cell migration (Fig. 3A and B). To determine the possible involvement of Mnk/eIF4E signaling pathways in the migration and invasion of TNBC cells, Mnk inhibitors (CGP 57380 and cercosporamide; 10 μmol/L each) were also used. Our results indicated that treatment of TNBC cells with Mnk inhibitors also inhibited cell migration, albeit significantly less effective than VNLG-152 (compared effect of 2.5 μmol/L of VNLG-152 *versus* Mnk inhibitors at 10 μmol/L; Fig. 3A and B). This data signifies the involvement of Mnk/eIF4E signaling pathway in TNBC cells migration. The inhibitory effect of VNLG-152 on cell migration and invasion was further confirmed by the PET membrane method (Supplementary Fig. 2) and matrigel invasion assay (Fig. 3C and D).

Recent reports have indicated Mnk-mediated eIF4E phosphorylation on serine 209 to be chiefly responsible for cellular transformation in many tumors including that of breast cancers. Mnk/eIF4E pathway is also reported to be indispensable for cellular proliferation, invasion and apoptosis evasion [10, 13]. To further support the involvement of Mnk in downstream oncogenic event of cell proliferation, we evaluated the expression of Mnk 1, peIF4E and cell cycle regulatory proteins in BC cells upon treatment with siRNA sequence of Mnk1. We found that addition of Mnkl siRNA not only resulted in strong knockdown of Mnk1, but also a significant decrease in the expression of downstream cell cycle regulatory proteins (Supplementary Fig. 3). Since the present study has demonstrated that RRs (VNLG-152) strongly inhibit cell proliferation, apoptosis evasion, invasion and migration in BC cells by modulating several proteins including that of cyclin D1 (Fig. 1D), and Bcl-2 (supplementary Fig. 4) that are predominantly regulated by the Mnk/eIF4E pathway, we hypothesized that RRs might disrupt these downstream oncogenic events primarily by inhibiting Mnk/eIF4E pathway. We therefore set forth to evaluate the effects of RRs on Mnk and eIF4E protein translational machinery.

### Inhibition of Mnk by RRs block eIF4E phosphorylation in breast cancer cells

**Figure 4:**
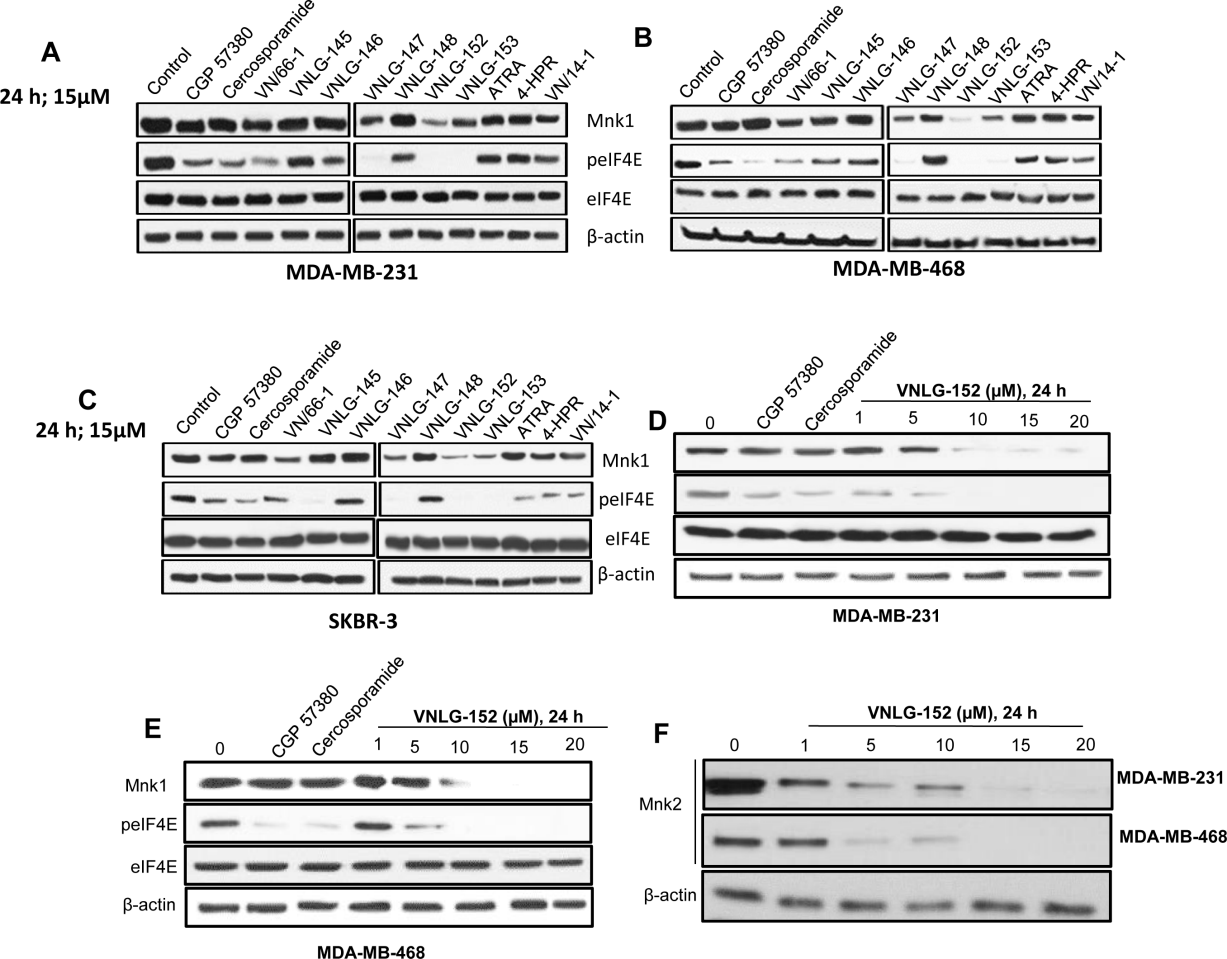
RRs reduce Mnk expression and eIF4E phosphorylation in breast cancer cells Equal protein concentrations from MDA-MB-231 (A) and MDA-MB-468 (B) and SKBR-3 (C) cells treated for 24 h with RRs, CGP57380 and cercosporamide (15 μmol/L) were separated by SDS-PAGE and western blots probed with antibodies to Mnk1 and serine 209 phosphorylated eIF4E. Companion blots were probed for total eIF4E. Dose response analysis for the inhibition of Mnkl and 2, peIF4E and eIF4E protein by RRs on MDA-MB-231 (D, F) and MDA-MB-468 (E, F) cells treated with VNLG-152 at indicated concentration and CGP 57380 or cercosporamide (15 μmol/L) for 24 h. Vehicle treated cells were included as a control and all blots were reprobed for β-actin for loading control.

To first check if RRs had the ability to target Mnks and peIF4E proteins, we probed the effect of RRs in comparison with ATRA, 4-HPR and the Mnk inhibitors-CGP57380 and cercosporamide on the expression of Mnk1 and p-eIF4E^ser209^ in BC cells by western blotting. We observed that 24 h treatment of well-established BC cells (MDA-MB-231, MDA-MB-468 and SKBR-3) with 15 μM of RRs reduced the expression of Mnk1 as well as phosphorylated eIF4E. However, no notable effect was observed in the expression of total eIF4E upon RRs treatment. Among the RRs tested, VNLG-152 exhibited highest degree of potency in down regulating both Mnkl and p-eIF4E (Fig. 4A, B and C). RRs also showed robust down-regulation of Mnkl and p-eIF4E than the clinically relevant retinoids (ATRA and 4-HPR), and Mnk inhibitors (CGP57380 and cercosporamide). We note that cercosporamide is currently in advancing preclinical studies by researchers at Lilly in view of clinical trials [33]. In addition to Mnkl, Mnk2 also phosphorylates the cap binding eIF4E at ser209, albeit to a lesser extent. We next performed a dose dependent analysis to determine the optimum concentration for the lead compound (VNLG-152) to induce Mnk degradation and inhibit eIF4E phosphorylation. We found that in both MDA-MB-231 and MDA-MB-468 cell lines, VNLG-152 exerted a dose dependent Mnk inhibition with maximal effect at 20 μmol/L (Fig. 4D, E and F). To further confirm whether the ability of VNLG-152 to reduce cell viability in TNBC cells at 2 μM (GI_50_) was due to downregulation of Mnks, we next performed a time dependent analysis by treating TNBC cells with VNLG-152 (2 μM) up to 72 h. As shown in supplementary Fig. 5, treatment with VNLG-152 induced a time dependent downregulation of both Mnks and p-eIF4E signifying that MnK downregulation contributed to the antiproliferative effects of VNLG-152 in TNBC cells.

To further explore the effect of RRs on the upstream Mnk kinases, we examined the expression of p-Erk and p-p38 by western blotting. Our results indicated that (15 μM, 24 h) treatment of BC cells with RRs did not induce any significant change in the expression levels of either p-Erk or p-p38, signifying that effect of RRs on eIF4E phosphorylation are not mediated via the upstream kinases of Mnk (supplementary Fig 6A and B). Alternatively RRs may act via the protein phosphatase 2A, (PP2A) an enzyme chiefly responsible for dephosphorylating Mnk1 and eIF4E [38], to inhibit Mnk/eIF4E pathway. To address this, we analyzed the effect of RRs on the expression of PP2A in MDA-MB-231 and MDA-MB-468 cells. Our results revealed that 24 h treatment of BC cells with 15 μmol/L of RRs did not induce any remarkable changes in the expression of PP2A, indicating that RRs-mediated inhibition of eIF4E phosphorylation was not the result of depletion of PP2A (Supplementary Fig. 6C and D). Recent studies have suggested that inhibition of eIF4E translation via rapamycin leads to an increase in p-Akt [39]. We therefore evaluated the effects of RRs on the expression of Akt and p-Akt another potential mediator of Mnk/eIF4E pathway. We found that RRs treatment did not cause any changes in the levels of Akt and p-Akt proteins in all 3 breast cancer cell lines (Supplementary Figure 7).

### Inhibition of Mnk/eIF4E pathway by RRs is independent of in vitro Mnk kinase activity

Because RRs cause a significant reduction in the expression of Mnkl, we further screened RRs in comparison with staurosporine, a broad spectrum kinase inhibitor for *in vitro* Mnk1/2 kinase profiling assay to determine whether the RRs inhibit Mnk1/2 kinase activities *(studies conducted by CRO)* [40]. We found that although the reference compound, staurosporine inhibited both Mnk1/2 activities, RRs did not have any inhibitory effects on either Mnkl or 2 in the range of 0.1 nmol/L −10 μmol/L (supplementary Table 1). These findings suggest that RRs do not have any considerable effects in inhibiting Mnk kinase activity *in vitro* but rather cause its degradation to mediate the inhibitory effect.

### Mechanism of RRs mediated Mnk1 degradation and the importance of Mnkl in eIF4E phosphorylation

**Figure 5:**
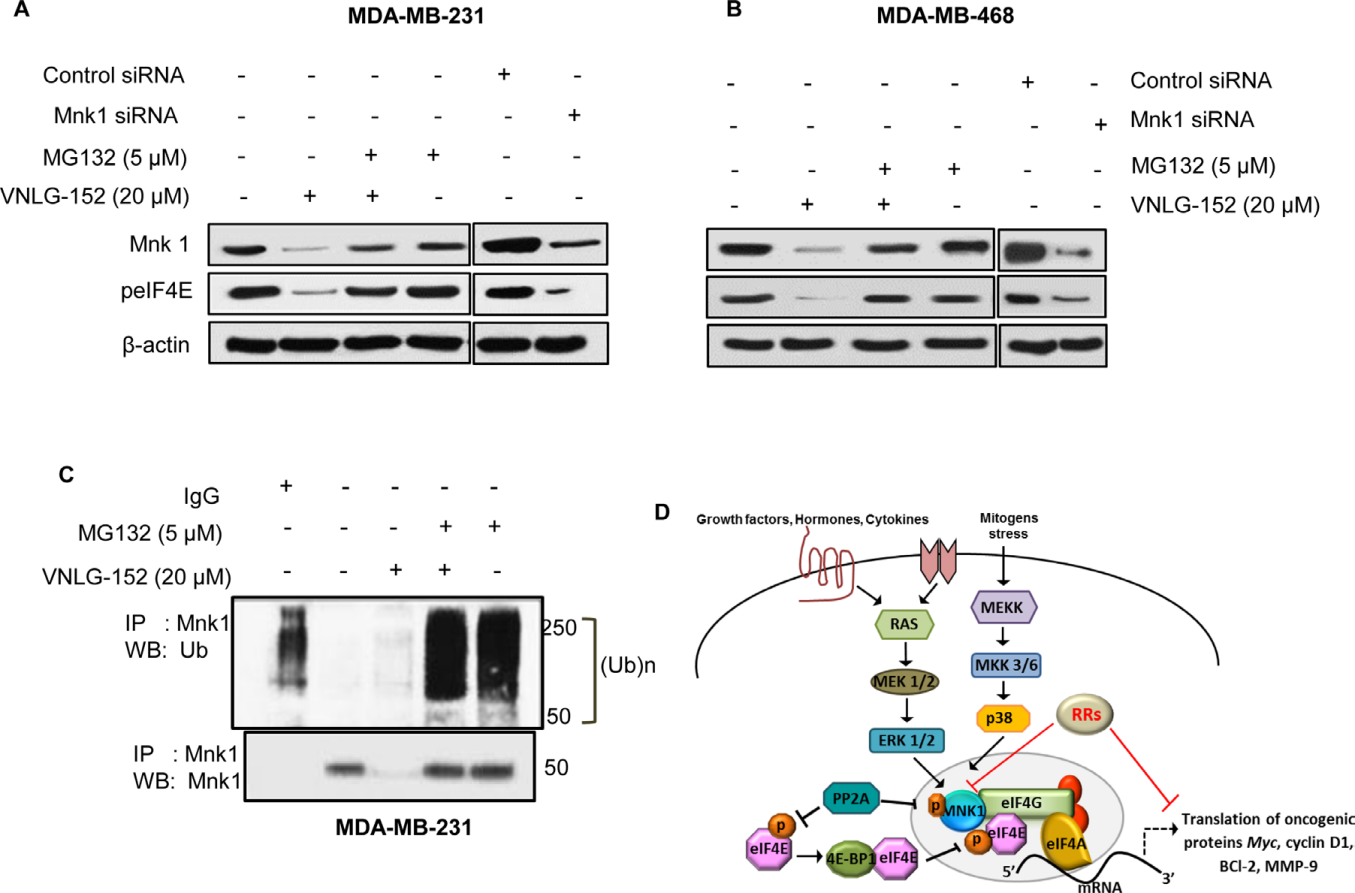
VNLG-152 induced degradation/polyubiquitination of Mnk protein and schematic model of MNK mediated eIF4E activation and inhibition by RAMBA retinamides (VNLG-152) MDA-MB-231 (A) and MDA-MB-468 (B) cells were treated with 20 μmol/L of VNLG-152, 5 μmol/L of MG-132, and combinations for 24 h. Mnk protein was immunoprecipitated with Mnk antibody (mouse) and the precipitated protein was subjected to western blot analysis with anti-ubiquitin antibody (Ub) (C, upper panel). The same blot was used to detect Mnk protein with anti-Mnk (rabbit) antibody after stripping (C, lower panel). Knockdown of Mnk1 by transfection with siRNA against (Mnk1) or its scramble control blocks eIF4E phosphorylation in MDA-MB-231 (A) and MDA-MB-468 (B) cells. All blots were reprobed with β-actin for equal protein loading and transfer. The data are representatives of two independent experiments. D: Activation of Ras/Erk pathway or p38 MAPK downstream of cytokine or stress stimuli results in activation of Mnk1 via phosphorylation. Activated Mnk subsequently binds to eIF4G and phosphorylates eIF4E within the eIF4F complex, resulting in the translation of certain mRNAs vital for oncogenic transformation. Conversely, PP2A can directly dephosphorylate Mnk and eIF4E proteins, leading to increased association of eIF4E with 4E-BP1 protein and reduced eIF4E translational machinery. RAMBA retinamides exert their inhibitory mode of action by inducing Mnk1 degradation subsequently reducing eIF4E phosphorylation and eIF4E driven cap dependent mRNA translation.

To determine whether the decrease in eIF4E phosphorylation by RRs is dependent on Mnk, we examined the effect of lead compound VNLG-152 in combination with MG-132, a proteasome inhibitor in MDA-MB-468 and MDA-MB-231 cells. We found that treatment of both these cell lines with 20 (μmol/L of VNLG-152 for 24 h degraded Mnkl significantly compared to that of control and MG-132 alone treated cells. In cells that were co-treated with VNLG-152 (20 μmol/L, at 16 h) and MG-132 (5 μmol/L at 8 h), Mnkl degradation was rescued, thus confirming that VNLG-152 induces Mnkl degradation to inhibit eIF4E phosphorylation (Fig. 5A and B). VNLG-152 induced Mnk degradation was further confirmed by polyubiquitination. The polyubiquitination was detected by immunopecipitation of cell lysates with Mnkl, followed by Western blot analyses with anti-ubiquitin antibody (Fig 5C).

To further support the role of Mnkl in eIF4E phosphorylation, we assessed the expression of Mnk1 and peIF4E in BC cells upon treatment with siRNA sequence of Mnkl. We found that addition of Mnk1 siRNA resulted in robust knockdown of Mnk1 and a decrease in the expression of downstream peIF4E protein. siRNA mediated Mnk1 reduction also resulted in a proportionate decrease in the amount peIF4E protein that matched the decrease achieved by VNLG-152 on Mnkl and pIF4E proteins confirming that the mechanism underlying VNLG-152 inhibitory effect on Mnk/peIF4e pathway involved Mnk degradation (Fig 5A and B).

## DISCUSSION

Majority of breast cancer related deaths are typically due to late diagnosis and resistance to therapies which result in rapid progression and metastatic spreading. In this study we have used *in vitro* breast cancer models to examine the anti-cancer effects of RRs and further our hypothesis that RRs mediated anti-cancer effects are in part mediated via inhibition of cap dependent mRNA translation. Our results demonstrate that in Her2 overexpressing and TNBC cells, RRs, especially, VNLG-152 disrupt translation machinery resulting in inhibition of protein translation and downstream oncogenic events.

Several studies in recent years have identified the oncoprotein eIF4E and its phosphorylated forms to be overexpressed in breast carcinomas [13, 17]. eIF4E is also identified as a poor prognostic marker in several breast cancer retrospective and prospective studies [17]. Phosphorylation of eIF4E at ser209 that occurs in response to various extracellular stimuli such as growth factors, hormones, and mitogens increases the affinity of eIF4E for the 52032; cap of mRNA and influences its entry into translation initiation complex. The best candidate for eIF4E phosphorylation is the mitogen-activated protein kinase (MAPK)-activated protein kinase Mnk1. Mnkl physically associates with eIF4F and directly phosphorylates eIF4E. In addition to Mnk1, Mnk2 is also recognized to phosphorylate eIF4E but to a lesser extent. Both Mnk1 and 2, particularly Mnk1, undergo activation via phosphorylation by extracellular signal-regulated kinase (ERK) or p38 mitogen-activated protein (MAP) kinases. Mnk mediated eIF4E phosphorylation favors mRNA translation of proteins involved in cell proliferation and survival, and correlates with cancer cell proliferation [10, 11, 41]. Wendel *et al.* have also shown that the oncogenic potential of eIF4E is strictly dependent on Mnk mediated ser209 phosphorylation in a mouse lymphoma model [32]. In a recent study, the antifungal agent, cercosporamide was reported to inhibit Mnk and block phosphorylation of eIF4E both *in vitro* and *in vivo* [33]. While Mnk function and eIF4E phosphorylation are considered indispensable for malignant transformation, Ueda *et al.*, demonstrated that Mnk mediated eIF4E phosphorylation are not essential for normal organismal development [42]. Thus, targeting Mnk/eIF4E pathway that is critical for cancer cell growth, survival and tumor progression is an ideal and attractive strategy for therapeutic intervention of breast cancers.

In this study, we have identified potential therapeutic agents that prevent Mnk-dependent phosphorylation of eIF4E in different breast cancer cell subtypes including TNBC for which targeted therapies are currently not available. The biological significance of this phenomenon is indicated by enhanced cell death and inhibition of cell growth, migration and invasion in RRs (VNLG-152) treated cells where eIF4E phosphorylation is prevented by Mnk1 degradation. We report here that RRs significantly induce Mnk1 degradation that results in the loss of Mnk1's ability to phosphorylate eIF4E at ser209 without affecting total eIF4E levels. These results are consistent with the recent findings by Konicek *et al*., on Mnk inhibition and eIF4E phosphorylation to inhibit tumor growth and induce cell death *in vitro* and *in vivo* [33]. Importantly, we note that RRs display more potent activity in depleting Mnkl and peIF4E proteins compared to the other clinically relevant retinoids (ATRA and 4-HPR) and Mnk inhibitors (cercosporamide and CGP57380). The ability of the RRs to induce Mnk degradation and block eIF4E phosphorylation may further block the eIF4E mediated cap dependent translation in breast cancer cells. Conceptually, Mnkl degradation provides a potential mechanism for antagonizing non-kinase dependent Mnk1 functions.

Mnk degradation and eIF4E phosphorylation are the foremost focus of this study as eIF4E is the utmost substrate of Mnk, particularly with respect to malignancy [23, 41]. Mnk activation is usually a downstream consequence of ERK and/or p38MAPK activating kinases. Besides these upstream kinases, Mnk and eIF4E may also be regulated by protein phosphatase 2 (PP2A) via dephosphorylation [23, 38, 41]. Our results indicate that RRs do not have any significant effects on ERK/p38MAPK, Akt or PP2A, and that RRs mediated Mnk downregulation apparently involves ubiquitin dependent degradation that was confirmed with studies using proteasomal inhibitor MG-132. We also found that breast cancer cells that were treated discretely with Mnk1 siRNA and RRs resulted in a similar pattern of results on Mnk1 and peIF4E expression, signifying that RRs function in a manner similar to that of Mnk1 siRNA that mediate Mnk1 knockdown. Unlike most regulatory mechanisms, protein degradation is intrinsically irreversible and RRs induced Mnk degradation can result in a rapid, complete and sustained termination of downstream eIF4E cap dependent translational process in breast cancer cells which eventually results in inhibition of cell growth, colony formation and tumor invasion as well as induction of caspase dependent cell death. Several studies have reported that the proteins cyclins A, B, D, and E, CDK inhibitor p27, p21, transcription factor E2F, retinoblastoma (Rb), and tumor suppressor p53 are regulated by proteasome-mediated proteolysis. The blockage of cell cycle progression with proteasome inhibitors is currently used against various forms of cancer [43]. Figure 5D represents the overall effects of RRs on Mnk, eIF4E, upstream activating kinases and downstream oncogenic events in breast cancer cells.

In conclusion, the data provided in this manuscript show that RRs degrade Mnkl and block eIF4E phosphorylation to possibly promote cell death and inhibit cell growth survival and invasion in breast cancer cells. To the best of our knowledge, ours is the *first preclinical proof of evidence for agents capable of inducing Mnkl degradation in cancer cells*. Furthermore, we are unaware of any reported *small-molecule Mnk degrading agents (MNKDAs)* capable of inducing depletion of eIF4E phosphorylation and translational machinery. Reports from other laboratory have demonstrated that CGP57380 and cercosporamide block eIF4E phosphorylation by inhibiting Mnk activity rather than degrading Mnk [33, 34]. Significantly, unlike the Mnk inhibitors (CGP57380 and cercosporamide) and two clinically relevant retinoids (ATRA and 4-HPR) [35, 36], RRs show potent breast cancer cell growth inhibition and down-regulation of p-eIF4E. RRs mediated-Mnk1 degradation and inhibition of eIF4E phosphorylation observed in the present study is of significance considering the emerging attention in control of mRNA translation in general and the potent novel anticancer effects of Mnk degrading agents in particular. Our data support the notion that agents which degrade Mnk and block eIF4E mediated downstream events are likely to be superior to selective small molecule Mnk inhibitors such as cercosporamide and CGP57380 in the treatment of human cancers. Our data also highlight the potential utility of these compounds in investigating Mnk function compared to other commercially available Mnk inhibitors. Based on the well-established pharmacologic safety of the structurally related VN/66-1 synthesized previously by us [29] and 4-HPR [35], we envision that these new RRs will be nontoxic (*safe*) with excellent druglike properties.

Because RRs degrade Mnk1/2 in aggressive breast cancer cells, a rational drug development strategy could be used to further progress these compounds towards advanced preclinical and clinical testing. Moreover, reducing Mnk/p-eIF4E expression with novel RRs could also provide a therapeutic strategy for the treatment of additional aggressive cancers because Mnk1/2 and eIF4E/p-eIF4E expressions are reported to be up-regulated during progression of all types of solid tumors [9] and hematologic cancers [44].

## MATERIALS AND METHODS

### Cell culture treatment and Western blotting

MDA-MB-231 and MDA-MB-468 cells were purchased from ATCC and cultured in the recommended media supplemented with 10% FBS. Cells were plated 1 day prior to treatment. Mnk inhibitors (CGP5730 and cercosporamide), clinically relevant retinoids (ATRA and 4-HPR) were treated at 15 μmol/L and RAMBA retinamides were treated at indicated concentration and time periods and incubated at 37 °C in 5% CO_2_ Protein lysates were harvested in RIPA lysis buffer (sigma). Western blotting was done as described previously [30] with the following antibodies: p-eIF4E, Mnk1/2, total eIF4E, PP2A, p-p38, p-ERK, CDK2 and 4, cyclin B1, cyclin D1 and D3, CDC25C, Bcl-2, β-actin (Cell Signaling), mouse and rabbit horseradish peroxidase (HRP)-conjugated secondary antibodies (Santa Cruz Biotechnology).

### Cell growth and colony formation assays

Cell growth inhibition assay was performed as described previously [28]. For cell growth experiment, cells were treated with the RRs for 6 days. MTT assay was performed at the end of the experiment. Calculations of combination indices were done using the Calcusyn program (Biosoft, Cambridge, United Kingdom). For colony formation assay, cells were plated 1000 per well in complete media in six-well plates and allowed to adhere for 24 h. The next day cells were treated with (VN/14-1, ATRA, CGP57380, cercosporamide and 4-HPR) and RRs (10 μmol/L). After 24 h compound containing media were removed, and cells were allowed to form colonies in complete media. Approximately 2–3 weeks later the colonies were fixed, stained with 0.5% crystal violet (sigma) for 30 min and counted manually. Results represent the mean ± standard deviation of three independent experiments.

### Cell cycle analysis

Cell cycle analysis was performed as described previously [30, 31]. 0.3 × 10^6^ cells were seeded in a 6-well plate and treated for up to 72 h with VNLG-152 (2 μM). Cells were stained with 40 mg/mL PI prior to analysis by a FACS calibur instrument (Becton Dickinson, San Jose, CA, USA) employing the Cell Quest Software.

### MNK kinase assay

*In vitro* profiling of the Mnk1 and Mnk2 kinase was performed at Reaction Biology Corporation (Pennsylvania) using the “HotSpot” assay platform. Concisely, specific kinase/substrate pairs along with required cofactors were prepared in reaction buffer; 20 mmol/L Hepes pH 7.5, 10 mmol/L MgCl_2_, 1 mmol/L EGTA, 0.02% Brij35, 0.02 mg/ml BSA, 0.1 mmol/L Na_3_VO_4_, 2 mmol/L DTT, 1% DMSO. 10 μmol/L concentrations of compounds were disseminated into the reaction followed ~20 min later by addition of a mixture of ATP (Sigma) and ^33^P ATP (PerkinElmer. Reactions were carried out at 25 °C for 120 min, followed by spotting of the reactions onto P81 ion exchange filter paper (Whatman). Unbound phosphate was removed by extensive washing of filters in 0.75% phosphoric acid. After subtraction of background derived from control reactions containing inactive enzyme, kinase activity data were expressed as the percent remaining kinase activity in test samples compared to vehicle (dimethyl sulfoxide) reactions.

### Immunoprecipitation and detection of ubiquitination of Mnk protein

MDA-MB-231 and MDA-MB-468 cells were treated with VNLG-152 (20 μ mol/L) and MG-132 (5 μ mol/L) and combination thereof for 24 h. MG-132 was added 8 h prior to the VNLG-152. Treated and control cells were lysed with IP/lysis buffer (20 mmol/L Tris pH = 7.4, 150 mmol/L NaCl, 1% triton X-100, 0.1%NP40, 1 mmol/L EDTA, 1 mmol/L PMSF, 1X protease inhibitors cocktail). 0.5 mg of total cell lysates were pre-cleared with 20 mL of protein A/G sepharose beads (Santa Cruz), for 45 min and pelleted for 1 min at 13,300 rpm. Supernatants were collected and incubated with 1 ug of polyclonal antibody per 500 ug of total protein in immunoprecipitate. Protein lysate-antibody complex were rotated for 12 h at 4 °C and beads added for an additional 1 h. Complexes were centrifuged in microcentrifuge at 4°C and the supernatant was discarded. Beads were washed with 3X IP/lysis buffer and were re-suspended in 2X SDS sample loading buffer and boiled at 99 °C for 5 min. Samples were then separated in SDS-PAGE and used for Western blot analysis using ubiquitin antibodies after stripping of the membrane for Mnk.

### Small interfering RNA experiment

For siRNA transfection, cells at a concentration of 2.5 or 5 × 10^4^ cells/mL were incubated for 24 h in six-well plates in culture medium. The cells were then transfected with Mnkl siRNA and non-targeting siRNA (purchased from Ambion) for 72 h in the presence of oligofectamine (Invitrogen). Protein silencing was further confirmed by immunoblot analysis.

### Cell apoptosis assay (acridine orange/ethidium bromide (AO/EB) dual staining; and cell death detection ELISA assay)

In each of this assay, cells were treated with indicated concentrations of the compounds for 72 h. Assessment of the apoptotic potential by Cell Death Detection ELISA assay was done as per protocol of the manufacturer (Roche diagnostics). For AO/EB dual staining, acridine orange (0.1%) and ethidium bromide (0.2%) in PBS were added to the cells and incubated at 37 C and 5% C0_2_ for 30 min. Cells were then immediately analyzed using Nikon TE2000 fluorescence microscope.

### Scratch motility (wound-healing) assay

The anti-migratory efficacy of RRs was examined using the well-established wound healing assay [45], MDA-MB-231 and MDA-MB-468 cells were plated in a 24 well plate at 5 × 10^5^ cells per well and allowed to form a confluent monolayer for 24 h. Cells were made dormant by pretreating with 0.5 μmol/L mitomycin C for 2 h to ensure that wounds are filled due to cell migration and not by cell proliferation [46]. Subsequently, the monolayer was scratched with a pipette tip, washed with media to remove floating cells, and photographed (time 0). Cells were then treated in the presence or absence of 2.5 and 10 μmol/L concentrations of indicated compounds. Experiment was terminated as soon as wound was completely filled in vehicle treated controls. Cells were then photographed again using Nikon TE2000 microscope at three randomly selected sites per well.

### Migration assay

The migration assay was conducted using polyethylene terephthalate (PET) membrane (8-μm pore size) tissue culture (TC) insert from Millipore. Briefly. TNBC cells were plated into the upper chamber of the TC insert containing serum free media, and the insert was placed into a well of a 24 well plate containing various compounds (10 μmol/L) including VNLG-152. The control well contained serum free media only. After 24 h, the top surface of the non-migrated cells were scraped with cotton swabs and the cells on the lower surface of the membrane (migrated cells) were fixed for 15 min with cold MeOH and stained with crystal violet. Cells that had migrated to the bottom of the membrane were visualized and counted using an inverted microscope. For each replicate (n = 3), cells in four randomly selected fields were counted and averaged.

### Invasion assay

Invasion assay for BC cells was performed similar to migration assay as described above, but in this assay the trans-well chambers coated with matrigel was used.

### Statistical analysis

All experiments were carried out in at least triplicates and are expressed as mean ± S.E. where applicable. Treatments were compared to controls using the Student's t-test with either GraphPad Prism or Sigma Plot. Differences between groups were considered statistically significant at P < 0.05.

## Supplementary Figures and Table




