# Supplementary material for: First Mnks degrading agents block phosphorylation of eIF4E, induce apoptosis, inhibit cell growth, migration and invasion in triple negative and Her2-overexpressing breast cancer cell lines
Source: Oncotarget. 2014 Jan 25;5(2):530–43. doi: 10.18632/oncotarget.1528 (PMC3964227; doi:10.18632/oncotarget.1528)
Supplement: Supplementary file 1 [file oncotarget-05-530-s001.pdf]

First MNK degrading agents block phosphorylation of eIF4E, induce apoptosis, inhibit cell growth, migration and invasion in triple negative and Her2-overexpressing breast cancer cell lines – Senthilmurugan et al

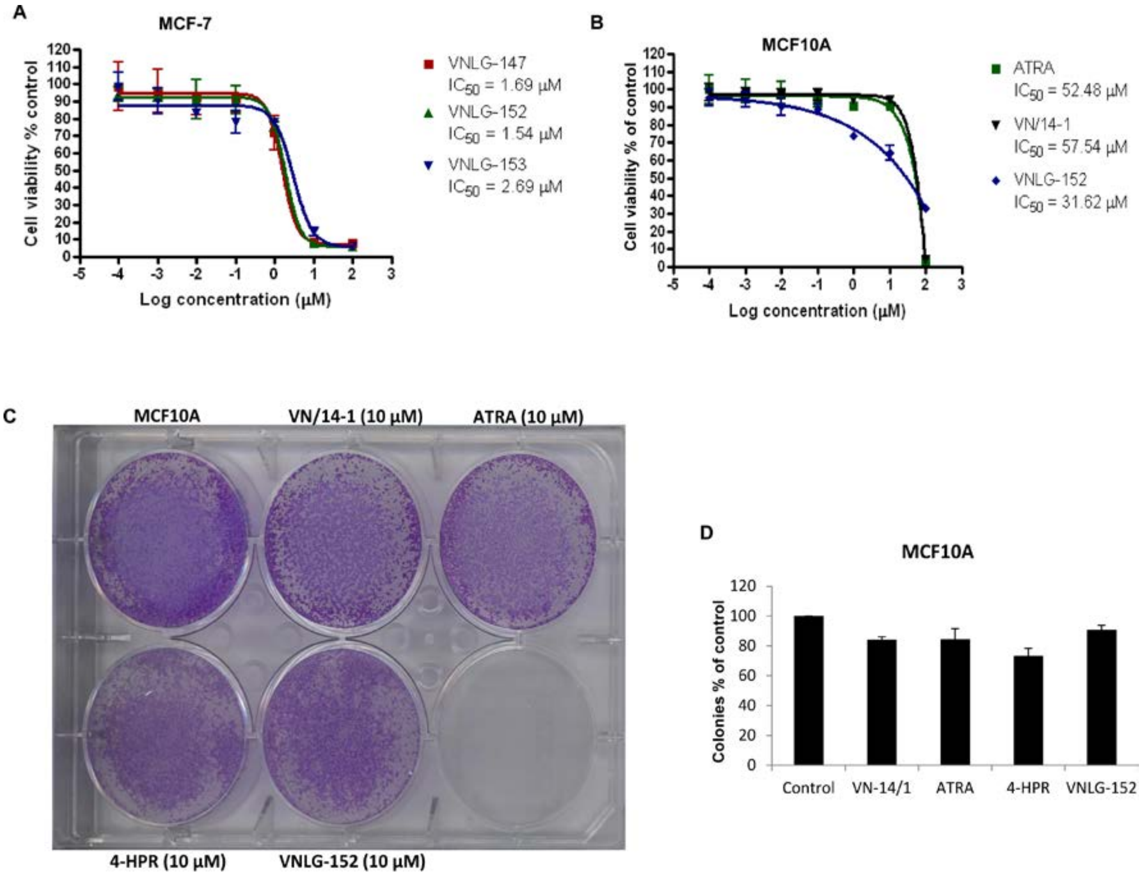

Supplementary Figure 1: Antiproliferative potential of RRs in MCF-7 and MCF10A cells (A) VNLG-147, 152 and 153 inhibits the growth of MCF-7. (B) Growth inhibitory effects of VNLG-152 on immortalized noncancerous MCF10A cell line. Curves generated from an MTT assay after 6 days exposure to indicated compound. Points: mean of replicates from 3 independent experiments; bars, SE. Solid line, best-fit sigmoidal dose response (variable slope). (C and D) MCF10A cells were treated with VN/14-1, ATRA, 4-HPR and VNLG-152 (10 µmol/L) for 14 days. Colonies were fixed with methanol and stained with crystal violet. Data represents the mean ± S.E from three independent experiments.

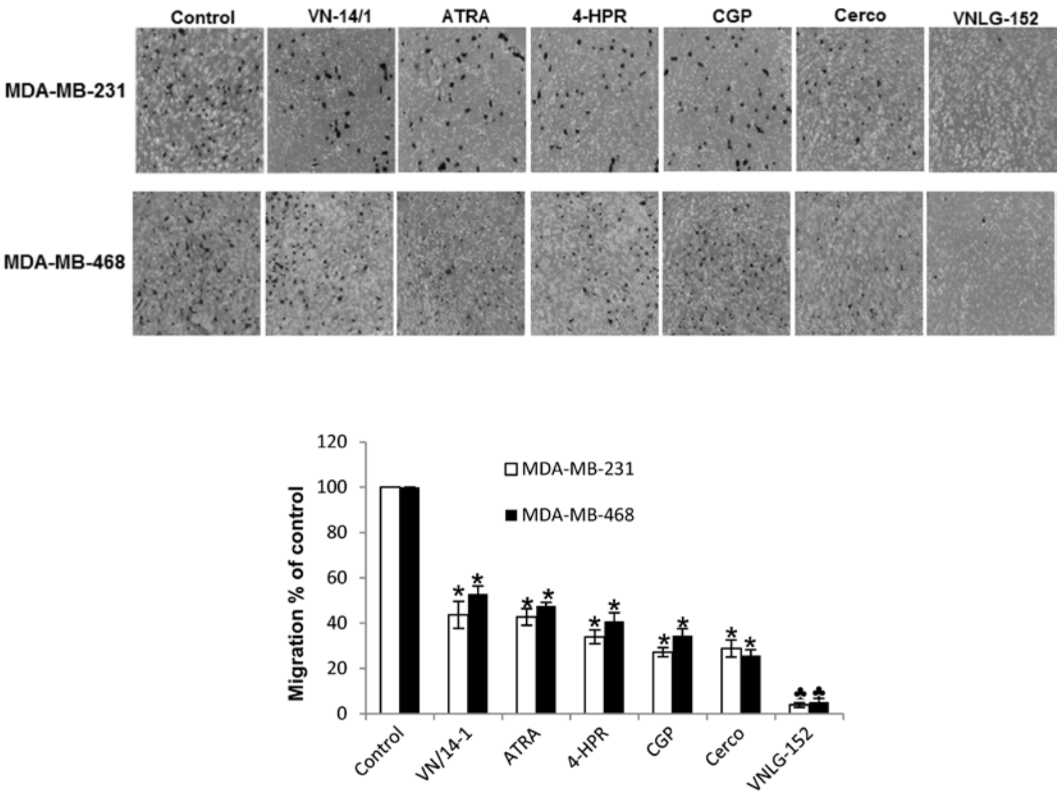

Supplementary Figure 2: VNLG-152 inhibits migratory potential of TNBC cells. Effect of the treatment of VNLG-152 (2.5 µmol/L) and indicated compounds (10 µmol/L) on breast cancer cell migration by PET membrane method for 24 h. Cells were seeded on boyden chamber and treated with VNLG-152 and indicated compounds. Dose selection was based on dose-dependent studies. Representative photomicrographs of PET membrane migration are shown at 100x magnification. Quantification of the number of migrating cells and the data are shown relative to vehicle treated control and the bars are means of three replicate determinations plus standard deviations. *, $P< 0.01$; ♣, $P<0.001$ compared with vehicle treated control.

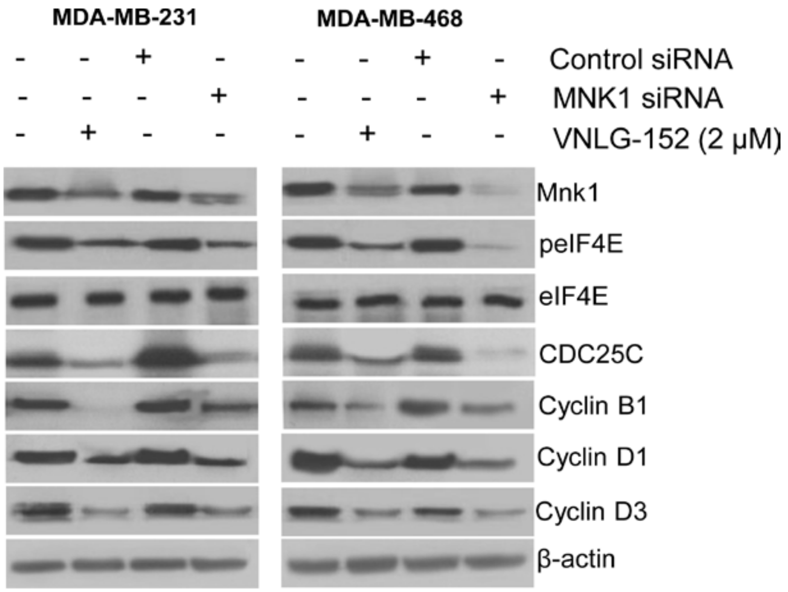

Supplementary Figure 3: Knockdown of Mnk1 by transfection with siRNA against (Mnk1) or its scramble control and cells treated with VNLG-152 (2 μM) blocks eIF4E phosphorylation and downstream cell cycle related proteins in MDA-MB-231 and MDA-MB-468 cells. All blots were reprobed with β-actin for equal protein loading and transfer. The data are representatives of two independent experiments.

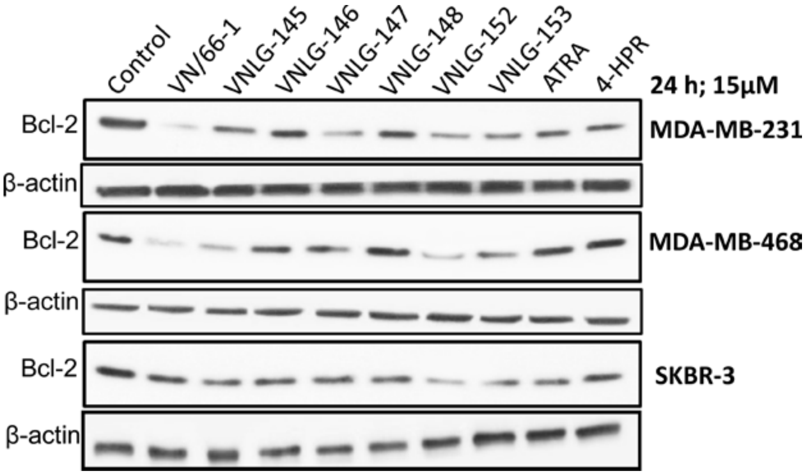

Supplementary Figure 4: Effect of RRs on the expression of anti-apoptotic Bcl-2 protein. MDA-MB-231, MDA-MB-468 and SKBR-3 cells treated for 24 h with RRs (15 μmol/L) were lysed and analyzed by western blotting with antibodies against Bcl-2. Vehicle treated cells were included as a control and all blots were reprobed for β-actin for equal protein loading and transfer.

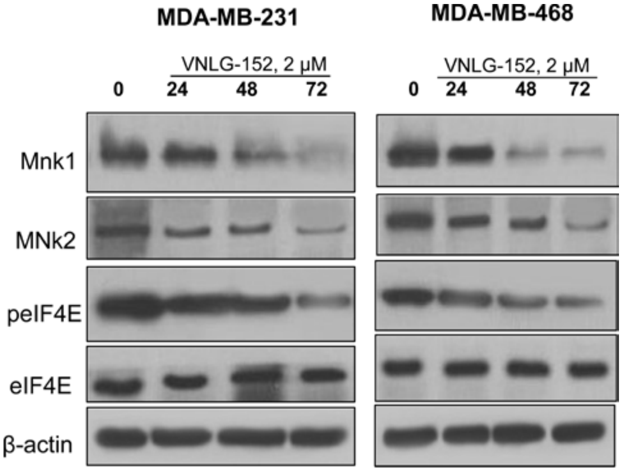

Supplementary Figure 5: Effect of VNLG-152 on Mnk and eIF4E in TNBC cells. Equal protein concentrations from MDA-MB-231 and MDA-MB-468 cells treated with VNLG-152 (2 μmol/L) for up to 72 h were separated by SDS-PAGE and western blots probed with antibodies to Mnk1, Mnk2 and p-eIF4E. Companion blots were probed for total eIF4E. Vehicle treated cells were included as a control and all blots were reprobed for β-actin for loading control.

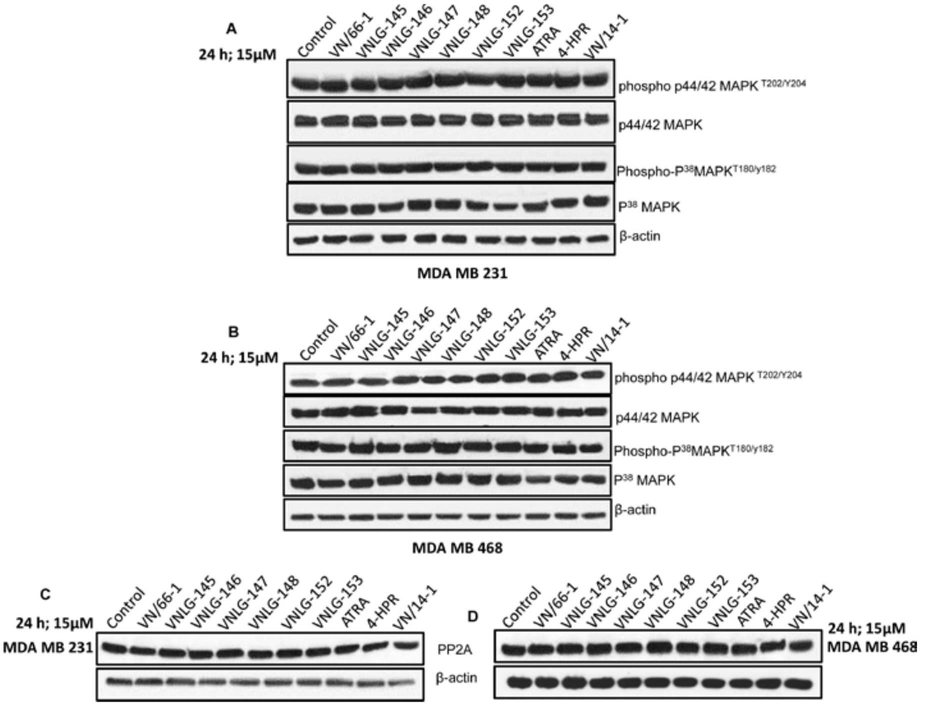

Supplementary Figure 6: Effect of RRs on upstream kinases of Mnk and PP2A proteins. MDA-MB-231 (A and C) and MDA-MB-468 (B and D) cells treated for 24 h with RRs (15 μmol/L) were lysed and analyzed by western blotting with antibodies against p-ERK, ERK, p-MAPK, MAPK and PP2A. Vehicle treated cells were included as a control and all blots were reprobed for β-actin for equal protein loading and transfer.

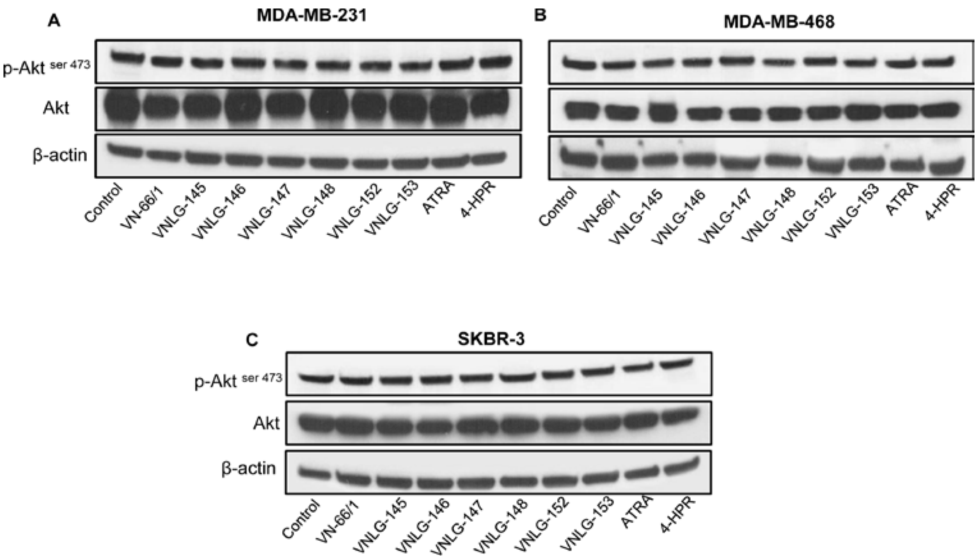

Supplementary Figure 7**:** Effect of RRs on Akt phopsphorylation. (A) MDA-MB-231, (B) MDA-MB-468 and (C) SKBR-3 cells treated for 24 h with RRs (15 µmol/L) were lysed and analyzed by western blotting with antibodies against p-Akt and Akt. Vehicle treated cells were included as a control and all blots were reprobed for β-actin for equal protein loading and transfer.
