# Supplementary material for: First Mnks degrading agents block phosphorylation of eIF4E, induce apoptosis, inhibit cell growth, migration and invasion in triple negative and Her2-overexpressing breast cancer cell lines
Source: Oncotarget. 2014 Jan 25;5(2):530–43. doi: 10.18632/oncotarget.1528 (PMC3964227; doi:10.18632/oncotarget.1528)
Supplement: Supplementary file 2 [file oncotarget-05-530-s002.pdf]

First MNK degrading agents block phosphorylation of eIF4E, induce apoptosis, inhibit cell growth, migration and invasion in triple negative and Her2-overexpressing breast cancer cell lines – Ramalingam et al

Supplementary Table 1: Effect of RRs on *in vitro* Mnk1 and Mnk2 kinases

	IC_{50} Values (nmol/L)*	
**Compounds**	**Mnk1**	**Mnk2**
**VN/14-1**	-	-
**VN/66-1**	-	-
**VNLG-145**	-	-
**VNLG-146**	-	-
**VNLG-147**	-	-
**VNLG-148**	-	-
**VNLG-152**	-	-
**VNLG-153**	-	-
**ATRA**	-	-
**HPR**	-	>10,000
**Staurosporine**	18.7	4.83

*Empty cells indicate no inhibition or compound activity up to 10,000 nmol/L that could not be fitted to an IC_{50} curve. Compounds were tested in 10-dose IC_{50} mode with 3-fold serial dilution starting at 10,000 nmol/L, control compound Staurosporine was tested in 10-dose IC_{50} mode with 3-fold serial dilution starting at 20,000 nmol/L, reactions were carried out at 1,000 nmol/L ATP.
